# Nanozyme-based sensors for detection of food biomarkers: a review

**DOI:** 10.1039/d2ra04444g

**Published:** 2022-09-15

**Authors:** Fareeha Arshad, Noor Faizah Mohd-Naim, Rona Chandrawati, Daniel Cozzolino, Minhaz Uddin Ahmed

**Affiliations:** Biosensors and Nanobiotechnology Laboratory, Faculty of Science, Universiti Brunei Darussalam Integrated Science Building Jalan Tungku Link Gadong BE 1410 Brunei Darussalam minhaz.ahmed@ubd.edu.bn minhaz.ahmed@outlook.com; PAPRSB Institute of Health Science, Universiti Brunei Darussalam Gadong Brunei Darussalam; School of Chemical Engineering, University of New South Wales (UNSW Sydney) Sydney NSW 2052 Australia; Centre for Nutrition and Food Sciences, The University of Queensland Australia

## Abstract

Nanozymes have piqued the curiosity of scientists in recent years because of their ability to demonstrate enzyme-like activity combined with advantages such as high stability, inexpensive availability, robust activity, and tunable properties. These attributes have allowed the successful application of nanozymes in sensing to detect various chemical and biological target analytes, overcoming the shortcomings of conventional detection techniques. In this review, we discuss recent developments of nanozyme-based sensors to detect biomarkers associated with food quality and safety. First, we present a brief introduction to this topic, followed by discussing the different types of sensors used in food biomarker detection. We then highlight recent studies on nanozyme-based sensors to detect food markers such as toxins, pathogens, antibiotics, growth hormones, metal ions, additives, small molecules, and drug residues. In the subsequent section, we discuss the challenges and possible solutions towards the development of nanozyme-based sensors for application in the food industry. Finally, we conclude the review by discussing future perspectives of this field towards successful detection and monitoring of food analytes.

## Abstract 2

We present a discussion on different types of sensors used in food biomarker detection and highlight recent studies on nanozyme-based sensors to detect markers like toxins, pathogens, antibiotics, growth hormones, metal ions, additives, small molecules, drug residues.

## Graphical abstract

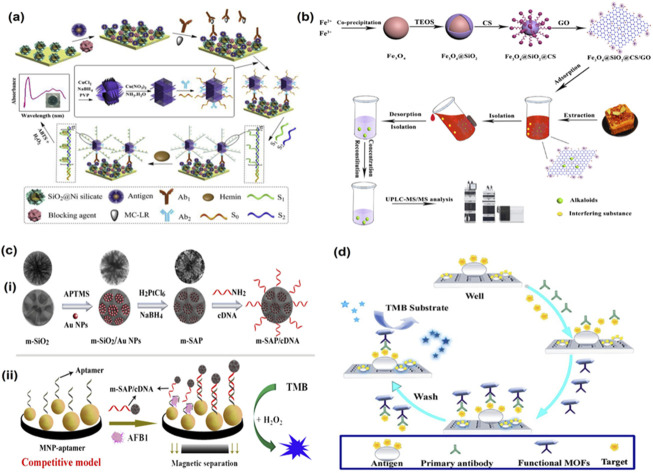

## 1. Introduction

Food safety is essential to protect consumers against any health risks and is one of the most fundamental research domains in biotechnology. In the recent decade, this area has received widespread attention from the scientific community in order to enhance food quality, both in developing and underdeveloped countries worldwide.^1,2^ Harmful elements found in many food items, for example toxins, antibiotics, hormones, and pathogens serve as a massive threat to human beings and animals alike.^3^ Not only does contaminated food harm living beings, but it also affects the economies of different countries around the world.^4^ Furthermore, food production has rapidly increased since the industrial and green revolution, primarily due to globalisation.^5^ As a result, there has also been a rise in food contamination that increases the chances of toxicants entering food items at every step of the production and transport process. Therefore, food safety is presently a fundamental challenge to the scientific community to protect living organisms from any health hazards arising from food contamination.

The World Health Organization has developed a set of rules and regulations that ought to be followed to uniformly monitor food safety and the resultant global challenges arising due to food contamination.^6,7^ In addition, it has also ordained the quality check of different food items to ensure its safety for the global population.^8^ Several techniques can successfully monitor food quality and safety. This includes chromatographic techniques, such as high-performance liquid chromatography, gas chromatography-mass spectrometry, and polymerase chain reaction. Although these methods allow for highly sensitive and selective detection of food contaminants, they are expensive, time-consuming, and require well-trained professionals to implement the procedure. This calls for alternate options that perform rapid, on-site detection and quantification of food contaminants. The previous decade has seen the development of impressive biosensors that allow for sensitive and selective detection of multiple biomarkers without extensive prerequisites.^9–15^

Conventional diagnostic techniques like immunoassays, agglutination, polymerase chain reaction (PCR), pathogen culturing among others, involve expensive and complex equipment, as well as require trained professionals to handle the system.^16^ In addition, they are costly to prepare, operate, and maintain; therefore, they are not user-friendly and not easily accessible to those in under-developed areas. Biosensors, especially those based on nanomaterials and nanoparticles (NPs), have several applications in medicine, environmental studies, and agriculture, among many others.^17^ In particular, nanomaterials with properties similar to enzymes, called nanozymes, have the potential to enhance the sensitivity of the biosensors due to signal amplification.^18,19^ The nanozyme like activity was first documented by Yan and colleagues in 2007 when they discovered the peroxidase-like activity of Fe_3_O_4_ NPs, which could catalyse the oxidation of peroxidase substrates in the presence of hydrogen peroxide (H_2_O_2_).^20^ Following this study, several classes of nanozymes have been discovered and studied that display different enzymatic properties when available alone or in conjugation with other ligands.^21^ Presently, many forms of nanozymes are available based on gold, graphene, platinum, zeolites, micelles, metalloproteins, supramolecules, and dendrimers.^8,10,22,23^ These are usually inexpensive and have considerably high stability. Hence, they are an exciting option to overcome the disadvantages of natural enzymes like low stability, lowered catalytic performance under changing conditions, additional steps required for purification of the enzymes, low reusability.^24^

Enzyme-based biosensors are commonly used due to their high sensitivity, selectivity, and specificity. Such sensors employ oxidation-reduction enzymes like glucose oxidase and horseradish peroxidase (HRP) to catalyze redox reactions by transferring electrons.^25^ When two or more enzymes are used in combination, the electrodes help in the simultaneous detection of multiple analytes.^26^ However, because of the high instability of enzymes, enzyme-based biosensors have a short shelf life.^25^ Therefore, nanozyme based biosensors are promising alternatives to such sensors. The application of nanozymes in biosensors is essential to developing inexpensive sensors that will help in the rapid diagnosis and monitoring of multiple biomarkers.

Nanozyme based sensors have promising potential in several fields like ensuring food quality and safety,^27^ disease detection and monitoring,^28^ and environment pollutant control.^19^ Nanozymes are basically nanomaterials that display enzyme like activities. They are the best alternatives to natural enzymes owing to their unique features like high stability, inexpensive, and easy storage.^29^ Nanozymes are often employed as electrode material in sensors or as tags that aid in signal amplification during the detection process. The resultant nanozyme-based biosensors display some exceptional advantages like shorter detection time, selective detection of the target analyte, better signal readout,^30^ and can be easily visualised with naked eyes, giving easy access to most users with uniform detection capability. Furthermore, nanozymes possess prominent features like high biocatalytic function, enhanced stability, rapid activity, comparatively inexpensive, and not affected by biological degradation activities.^31^ In addition, they have a large surface area to volume ratio and can be easily functionalised using a range of surface modification methods.^32^ Unique signal transduction properties, fluorescent activities, conductivity, and biocompatibility make nanozymes ideal for sensing food analytes and markers. Thus, nanozymes can be easily purified and modified compared to the naturally available enzymes. Also, the shape, diameter, and functional groups on the nanomaterials influence their enzyme-like activity.^33^ As a result, nanozymes are promising for developing analytical techniques for detecting food and clinical biomarkers.^17^ Hence, when combined with conventional methods like electrochemical and colourimetric methods, we can achieve sensing methods with high sensitivity and selectivity.

Some of the most significant advantages such sensors offer include functioning closely similar to natural enzymes during the sensing process, demonstrating high stability, and being active for longer durations. Moreover, such sensors display enhanced thermal stability, can be preserved with ease, and are reusable most of the time. Because of the ability of nanozymes in biosensors to give amplified detection signals, such uncomplicated biosensors are also used as a promising point of care devices for food analysis.^32,34^

So far, only a few articles have reviewed the data on nanozyme-based biosensors that aid food safety.^26,35–39^ Most of the review articles either provide an extensive discussion on the mechanisms of nanozyme-based biosensors^40^ or provide an overview of the biosensing strategies available for detecting food contaminants.^41^ For instance, Nguyen and Kim provided in-depth analyses of the nanomaterial-based colourimetric strategies to develop point-of-care devices for pathogen detection.^42^ In another instance, the same group extensively discussed nanomaterial-based colourimetric sensors for detecting various toxins.^43^ However, an in-depth discussion of nanozyme-based biosensors for the detection of various food biomarkers is rarely summarised. To better understand the various food biomarkers that can be analysed using biosensors, we have provided a comprehensive review of the recent developments in this field. We first discussed the types of sensors available to detect food biomarkers. We have then highlighted the mechanisms used to detect such biomarkers, followed by applications of nanozyme based biosensors in detecting different food biomarkers like toxins, pathogens, antibiotics, growth hormones, metal ions, additives, small molecules, and other drug residues. Next, we discussed the advantages and disadvantages of nanozyme-based sensors, followed by a brief discussion on nanozyme based sensors *versus* conventional sensors. Subsequently, we highlighted the challenges and possible solutions for the development of nanozyme based biosensors towards the detection of various food biomarkers. Finally, we presented the future perspectives of this rapidly evolving field. We believe that this article will help bridge the gap between nanobiotechnology and food sciences and provide an overview if the existent gaps in this field so that the scientific communities can collaborate and develop better and more efficient nanozyme based biosensors towards food safety.

## 2. Types of sensors for food biomarker detection

For quick and accurate analysis of different food biomarkers, several studies have been carried out using different sensing strategies in recent years. Electrochemical, piezoelectric, magnetic, optical, and photoelectrochemical sensors display unique advantages in detecting food analytes. Below, we have provided a quick overview of the different sensors used to analyse food biomarkers.

### 2.1. Electrochemical sensors

In such sensors, electrochemical signals are generated upon analysis of food components and are measured using potentiometric, conductometric, or amperometric methods.^44^ The analyte is detected at a close distance to the electrode surface, and the identification method used depends on the electrochemical properties of the electrode surface. Electrochemical sensors have significant advantages over the other sensors because of their high response rate, inexpensive availability, easy miniaturization, lowered detection limits and can perform detection even in minute concentrations of the sample.^45^ However, these sensors still have a few drawbacks that need to be addressed. For instance, they are susceptible to sample matrix effects, may not be as sensitive as the traditional methods, and have a shorter shelf life. Electrochemical sensors typically require periodic calibration. These limitations can be overcome by using nanomaterials.

Such sensors make the use of reference, counter, and working electrodes. Usually, the reference electrodes are made of silver chloride and are fixated far away from the biochemical reaction site; this is necessary to maintain a constant potential. The sensing electrode plays the role of a transduction source during the reaction. The auxiliary electrode allows the contact of the electrode surface and the electrolytic solution for current generation on the working electrode [9]. For successful biosensing activity, selecting an appropriate working electrode is essential. Recently, several works have focused on the development and modification of different electrode materials made from carbon elements,^46^ copper,^47^ gold,^48^ platinum,^49^ iron,^50^ cobalt,^51^ nickel, among others^52^ to improve the stability, selectivity, and sensitivity of the sensors. Furthermore, the miniaturisation of working electrodes has opened a promising path for developing small-sized sensors like the screen-printed electrode-based biosensing devices that need a minimal sample amount for sensing.^53^

Several studies have been done in the recent decade towards developing electrochemical biosensors for detecting various food biomarkers. For instance, Bagheri *et al.* developed a peroxidase mimicking Fe_3_O_4_ nanoparticles@ZIF-8 composite-based sensor to detect organophosphorus toxins in water and fruit juice samples.^54^ The developed nanocomposite displayed peroxidase mimicking activity that rapidly oxidized peroxidase substrates to give strong signals. The authors recorded a low detection limit of 0.2 nM and the detection range of the analyte was recorded to be between 0.5 and 500 nM. The researchers initially used this method to detect diazinon compounds and concluded that the method could be applied to detect the toxic organophosphorus toxins. In another study, reduced graphene oxide-based fluorescent aptasensor was developed to detect kanamycin in milk samples that could be detected at concentrations of as low as 1 pM.^55^

Apart from the electrodes, bioreceptors are essential components of a given biosensor. These receptors are biomolecular elements like enzymes, antibodies, or other protein molecules and nucleic acids that undergo a biochemical reaction with the target molecules to generate detectable signals for analysis. An ideal bioreceptor avoids interference with other molecules present in a complex sample mixture. Examples of these include DNA, proteins, aptamers that aid in the selectivity of the sensor. To enhance the sensing process during food analysis, attaching the receptors using different techniques like electrodeposition or introducing nanomaterial like nanozymes on the surface of working electrodes are promising steps towards developing sensitive and specific sensors.^44,56,57^ Thus, the appropriate immobilisation method used and the high specificity of the receptor molecules play a crucial role in improving the activity of electrochemical sensors for food analysis and food biomarkers detection.

### 2.2. Piezoelectric sensors

Piezoelectric sensors operate on the piezoelectric effect that occurs in specific materials, especially those that give an output voltage upon the introduction of any mechanical stress. At the same time, they also undergo the opposite effect; that is, they undergo mechanical changes upon being subjected to an electric voltage.^58^ Thus, the transducer acts as an actuator, and upon the application of electrical voltage, it emits ultrasound or vibrational waves in a given frequency range. This property is used to develop sensitive piezoelectric sensors for food analysis.^59–62^ In a study by Karaseva and colleagues, nanoparticulate molecularly imprinted polymers (NMIPs) were used as a molecular recognition element to develop piezoelectric chemical sensors to detect antibiotics, particularly penicillin.^63^ The researchers synthesized the NMIPs on the surface of the piezoelectric sensor through the precipitation polymerization method. The resultant sensor demonstrated sensitive and selective detection of penicillin G and ampicillin with a linear range of 0.1–0.5 μg mL^−1^ and 0.1–1.0 μg mL^−1^, respectively. The researchers further recorded the limits of penicillin G and ampicillin detection as 0.04 and 0.09 μg mL^−1^, respectively.

Such sensors are particularly advantageous because they do not require specific reagents or chemicals and detect based on affinity reactions alone. Compounds like aluminium phosphate, zinc oxide, quartz, polylactic acids, and other crystals that do not possess a centre of symmetry are ideal piezoelectric materials used in such sensors.^64^ However, piezoelectric sensors are appropriate for analyzing biomolecules with higher molecular weight due to their reduced oscillation frequency.^64^ Therefore, direct detection of biomolecules with lower molecular weight is not possible *via* this method and thus serves as a significant drawback of such sensors.

### 2.3. Optical sensors

Optical sensors allow for optical transduction of the target molecules and quantitatively analyse characteristics like amplitude, frequency, and phase using different probes. Compared to the traditional methods like chromatographic or spectrometric techniques, optical sensors are much more straightforward in terms of their configuration and thus are user friendly, inexpensive, and give rapid results. Furthermore, most optical biosensors work on the principles of surface plasmon resonance, total internal reflection fluorescence, waveguide-based SPR, integrated optical interferometers, among others. They, therefore, are promising towards the development of rapid, real-time, and label-free detection.

This was seen in the study by Wang and colleagues, who developed hemin-concanavalin A hybrid nanoflowers that displayed peroxidase-like activity.^65^ The as-developed sensor allowed the oxidation of 2,2′-azino-bis (3-ethylbenzthiazoline-6-sulfonic acid) diammonium salt (ABTS) in the vicinity of hydrogen peroxide to give green-coloured products. These nanoflowers developed thus played a crucial role during the colourimetric detection of foodborne *Escherichia coli* O157:H7 and demonstrated a low detection limit of 4.1 CFU mL^−1^ and linear range of 10^1^–10^6^ CFU mL^−1^. In another study, Lai *et al.* developed a colourimetric immunosensing platform based on MnO_2_ nanoflakes to sensitively detect aflatoxin B_1_.^66^ The researchers made the use of ascorbate oxidase/anti-aflatoxin B_1_ (AFB_1_) antibody labeled gold nanoparticles to detect AFB_1_ on AFB_1_-bovine serum albumin conjugated magnetic beads. When the target AFB_1_ were added to the sample, the target molecules competed with the conjugated AFB_1_-BSA on the magnetic beads for the labeled anti-AFB_1_ antibody attached with the gold nanoparticles. The authors recorded that with the increase in target AFB_1_, the absorbance decreased.

The major drawback of this method remains the high instrumentation cost.^67^ Regardless, optical biosensing options are particularly advantageous because they allow for quick and precise analyses of multiple food analytes and possess better flexibility against any electromagnetic interferences.^68^ Therefore, these biosensors permit the detection of several biomarkers in complex samples without prior treatments. Furthermore, when the analyte attaches to the sensing layer of the optical sensor *via* sorption or complex formation, the sensor's surface undergoes characteristic changes recorded by the sensor. Therefore, these sensors are particularly promising to detect food biomarkers like pathogens, drugs, pesticides, heavy metals, toxins to analyse the safety of the food.^69,70^

### 2.4. Photoelectrochemical sensors

Photoelectrochemical sensors usually comprise three major parts: a light source, a detection system, and the signal recorder. Photoactive materials are used to develop the working electrode to produce a photocurrent signal upon irradiating light.^71^ Like the previously discussed sensors, recognition receptors like antibodies, proteins, nucleic acids are essential for successful biosensing of the analytes. Furthermore, the characteristics of the photoactive component of the electrolyte can be modified by the target molecule, thereby causing changes in the photocurrent and the sensing result.

In a study by Liu *et al.*, the authors used a gold-modified TiO_2_ nanotube-based photoelectrochemical sensor to detect glucose molecules.^72^ The developed sensor was based on the strong charge separation efficiency and enhanced surface plasmon resonance of gold. The authors recorded a good sensitivity of 170.37 μA mM^−1^ cm^−2^ and a low detection limit of 1.3 μM. This sensor could be thus applied to detect glucose molecules in clinical and food samples.

Photoelectrochemical biosensors, especially the ones based on metal nanomaterials, have demonstrated a few drawbacks. These include enhanced oxidation capability causing analyte damage, destruction of the sensor because of photo corrosion, and the demand for high-energy light sources, especially for materials with large band gaps.^73^ However, unlike electrochemical sensing, in photoelectrochemical sensors, light is used as the excitation source leading to the generation of photocurrent, which translates into a signal produced by the sensor. Because of the varying energy levels of light compared to photocurrent, better sensitivity is noted in photoelectrochemical sensors.^74,75^ In addition, compared to the electrochemical biosensors, these sensors are less dependent on the applied potential, owing to their robust redox activity. Also, these sensors are relatively inexpensive, display high sensitivity, and can be used as a point of care device, thus making them great sensing devices for food analysis (Table 1).^76–78^

**Table 1:** Below sums up different nanozymes used in detection of food biomarkers *via* various types of biosensors

Sensing modality	Nanozyme used	Enzyme like activity displayed	Food biomarker detected	Sample matrix	Detection range	Limit of detection	Ref.
Optical sensor	Copper hydroxide	Peroxidase	Microcystin-LR (MC-LR)	MC-LR solutions; tap and lake water	7.03 × 10^−12^ M to 7.53 × 10^−8^ M	6.028 × 10^−9^ M	79
	Co_3_O_4_ magnetic nanozyme	Peroxidase	*Staphylococcus aureus*	Milk sample	10 CFU mL^−1^ to 10 000 CFU mL^−1^	8 CFU mL^−1^	80
	AuNP	Peroxidase	*Escherichia coli* O157:H7	Milk sample	1.25 × 10^1^ CFU mL^−1^ to 1.25 × 10^5^ CFU mL^−1^	1.25 × 10 CFU mL^−1^	81
	Gold nanoclusters	Peroxidase	Tetracycline antibiotics	Drugs and milk samples	1 × 10^−6^ M to 16 × 10^−6^ M	46 × 10^−9^ M	82
	Fe_3_O_4_ magnetic nanoparticles	Peroxidase	17β-estradiol	Milk, prawn, fish and chicken samples	0 M to 1.835 × 10^−9^ M	7.34 × 10^−10^ M	83
	Gold nanoparticles	Oxidase	*Listeria monocytogenes*	Pork sample	10 CFU mL^−1^ to 106 CFU mL^−1^	10 CFU mL^−1^	84
Electrochemical sensor	Gold nanoparticles	Peroxidase	*Escherichia coli*	Fruit juice	10 CFU mL^−1^ to 10^9^ CFU mL^−1^	∼100 cells per mL	85
	Graphene quantum dots	Peroxidase	*Yersinia enterocolitica*	Milk sample	1 CFU mL^−1^ to 6.23 × 10^8^ CFU mL^−1^	10^2^ CFU mL^−1^ to 10^6^ CFU mL^−1^	86
	Pt–Pd nanoparticles	Peroxidase	*Salmonella enteritidis* and *Escherichia coli* O157:H7	Milk and ice cream samples	10 to 107 CFU mL^−1^	∼20 CFU mL^−1^ and ∼34 CFU mL^−1^ for *S. enteritidis* and *E. coli* O157:H7 respectively	87
	CoFe_2_O_4_	Peroxidase	Kanamycin	Milk sample	1 × 10^−12^ M to 1 × 10^−6^ M	0.5 × 10^−12^ M	88
	TiO_2_/electro-reduced graphene oxide nanocomposites	Oxidase	Ponceau 4R and tartrazine	Detection in buffer solution	0.01 × 10^−6^ to 5.0 × 10^−6^ M	4.0 × 10^−9^ M and 6.0 × 10^−9^ M for ponceau 4R and tartrazine respectively	89
	TiO_2_ nanoparticles	Oxidase	Allura Red	Milk sample	0.3 × 10^−6^ M to 5.0 × 10^−6^ M	0.05 × 10^−6^ M	90
	GMP-Cu	Laccase	Sulfide	Baking soda, rock sugar, konjac flour and xylitol	0 M to 220 × 10^−6^ M	0.67 × 10^−6^ M	91
Piezoelectric sensor	Gold nanoparticles	Peroxidase	*Escherichia coli* O157:H7	—	10^2^ to 10^6^ CFU mL^−1^	1.2 × 10^2^ CFU mL^−1^	92,93
	Molecularly imprinted nanospheres	—	Penicillin G, ampicillin	—	0.1 to 0.5 μg mL^−1^ for penicillin G, and 0.1 to 1.0 μg mL^−1^ for ampicillin	0.04 for penicillin G and 0.09 μg mL^−1^ for ampicillin	63
Photoelectrochemical sensors	Au/TiO_2_NTs	Peroxidase	Glucose	—	1 to 90 μM	1.3 μM	72
	g-C_3_N_4_/QDs/rGO	Peroxidase	Sulfadimethoxine	Water samples	0.5 to 80 nM	0.1 nM	94
	NiTAPc-Gr/indium tin oxide	Peroxidase	Erythromycin	—	0.40 to 120.00 μmol L^−1^	0.08 μmol L^−1^	95
	TiO_2_ nanorod array sensitized with Eu(iii)-doped CdS quantum dots	Peroxidase	Chloramphenicol	Milk samples	1.0 pM to 3.0 nM	0.36 pM	96

## 3. Applications of nanozymes based biosensors for food biomarkers detection

Recently, several nanozyme based biosensors have been developed to detect different food biomarkers. This section discusses the applications of nanozyme based biosensors to detect toxins, pathogens, antibiotics, growth hormones, metal ions, additives, small molecules, and drug residues.

### 3.1. Toxins

Toxins are compounds that interfere with the body's functioning or cause severe damage like poisoning within the body. Sometimes, toxins even result in the death of the individual. For instance, mycotoxins are toxic compounds produced by fungi like *Fusarium*, *Aspergillus*, *Penicillium* and cause physiological damage in living beings.^97,98^ Likewise, other toxins like bacterial toxins, marine toxins, and plant toxins cause severe damage to the human body. These toxins enter the food chain *via* contaminated food products derived from plants or animals. Thus, it is necessary to develop high-sensitivity sensing methods to ensure that such toxins, even in trace amounts, do not go undetected during food analysis.

**Fig. 1:**
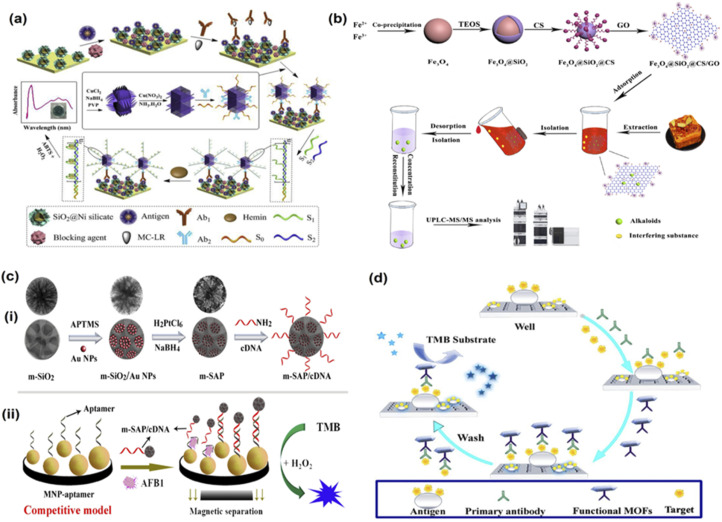
Nanozyme based sensors to detect toxins in food. (a) Schematic illustration of the immunosensor based on double-integrated mimic enzymes for the visual screening of microcystin-LR. Adapted with permission from ref. 79 copyright (2019) Elsevier. (b) The procedure for synthesis of Fe_3_O_4_@SiO_2_@CS/GO and its application in alkaloids. Adapted with permission from ref. 103, copyright (2020) Elsevier. (c) Schematic presentation of nanozyme and aptamer-based immunosorbent assay (NAISA): (i) the preparation process for m-SAP/cDNA and (ii) construction of NAISA method for AFB1 detection. Adapted with permission from ref. 99 copyright (2020) Elsevier. (d) Changes in the bioactivities of MOFs@Ab2 (black) and HRP@Ab2 (red) under different conditions. Adapted with permission from ref. 101, copyright (2021) Elsevier.

In a study by Wu *et al.*, an immunosorbent assay for the detection of aflatoxin B1 was developed using a combination of Fe_3_O_4_ magnetic nanoparticles (MNP) and mesoporous SiO_2_/Au–Pt (m-SAP) nanozymes as signal labels that displayed high catalase activity^99^ (Fig. 1). The authors used an aptamer to recognise aflatoxin B1 molecules with high specificity. The authors developed a nanozyme and aptamer-based competitive immunoassay model, in which the reaction steps involved three significant changes compared to conventional ELISA. As per the method, m-SAP acted as the enzyme label and MNP were attached to the lower part of the plate that functioned as the magnetic substrate. While an aptamer was employed that detected the target analyte and cDNA functioned as the signal tag. This method showed a low detection limit of 5 pg mL^−1^ and displayed a high specificity for the target analyte even in complex mixtures (peanut samples). Therefore, this method is particularly advantageous due to the simple action mechanism and high selectivity for the toxin. In another similar study for aflatoxin B1 detection, Hong and colleagues developed a magnetic relaxation method using gold nanoparticles.^100^ The sensing was based on self-assembly cascade signal amplification using AuNPs to detect the target analyte with high sensitivity. They developed a probe using AuNPs labelled with aflatoxin B1 antibody and initiator DNA, which allowed for triple cascade signal amplification, and this offered a high sensitivity to this sensing technique. The authors recorded a LOD of 0.453 pg mL^−1^ and displayed high specificity towards aflatoxin B1 detection. Similarly, in a closely related study by Xu and coworkers, an indirect competitive metal–organic framework (MOF) linked immunosorbent assay was developed *via* the hydrothermal method to detect aflatoxin B1 molecules in drink samples including peanut and soy milk samples^101^ (Fig. 1). The researchers synthesized the secondary antibody over the MOF surface using the covalent coupling method and then further developed the HRP@Ab_2_ using the same method. By studying the steady-state kinetics of the developed nanozyme, MOFs@Ab_2,_ the team discovered that the nanozyme demonstrated better peroxidase mimicking catalytic activity than HRP. The researchers added that the enhanced activity of the nanozyme could be because of its excellent hydrophilicity, which permits the nanozymes to be well-dispersed in the aqueous phase. This, therefore, allows the interaction of nanozymes and substrates in the same phase. In addition, the MOFs@Ab_2_ interacts with substrates with more active sites and pores, thereby allowing faster catalysis of substrates and nanozymes. This enhanced catalysis of the nanozymes thus allows for quick detection of aflatoxin B1. This method used functional MOFs to catalyse a chromogenic system and offered 20 times better LOD than conventional techniques like ELISA and therefore reduces the chances of false results. The LOD was recorded as 0.009 ng mL^−1^, and the linear range was noted to be 0.01 to 20 ng mL^−1^.

Nanozyme based sensing strategies for detecting bacterial and plant toxins have also been developed. For instance, Shlyapnikov and coworkers developed a micro assay-based immunoassay was developed simultaneously to detect five different bacterial toxins.^102^ The assay involves three primary steps. First, the toxins get collected over the antibody microarray, followed by tagging antigens with secondary biotinylated antibodies. Finally, the biotin labels get detected using streptavidin-coated magnetic beads in shear flow. When electrical and magnetic fields are applied in a single flow cell, the microarray beads get optically detected. The bacterial toxins detected by this sensor included *E. coli* heat-labile toxin, cholera toxin, and *S. aureus* toxins. The LOD recorded was 0.1–1 pg mL^−1^ for water, and 1 pg mL^−1^ in food samples and the whole process took less than 10 minutes. Liu *et al.* developed an immunosensor based on Cu(OH)_2_ nanozyme and G-quadruplex/hemin DNAzyme to detect microcystin-LR, another bacterial toxin^79^ (Fig. 1). The Cu(OH)_2_ nanozyme captures the secondary antibody and the substrate to load DNAzymes to visually see the target analyte within the range of 0.007 to 75 μg mL^−1^ and a LOD of 6 ng mL^−1^. Thus, nanozyme based biosensing techniques demonstrate high sensitivity and specificity and hence have a promising potential for detecting various toxins from food analytes.

### 3.2. Pathogens

Pathogens like bacteria and virus particles in food items have become a central matter of concern in the past decade. Once these pathogens enter food items, they may multiply and produce multiple toxic metabolites. Among these, *E. coli* O_157_:H_7_ and human norovirus are responsible for most health concerns due to spoilt food consumption. Furthermore, foodborne pathogens cause severe infections like food poisoning, and thus their rapid detection is necessary to ensure food safety. Though conventional methods like ELISA have helped detect different food biomarkers, including pathogens, they have been relatively time-consuming, expensive, and demonstrated low sensitivity. Therefore, sensing strategies based on nanozymes can prove advantageous towards detecting pathogens for food safety.

**Fig. 2:**
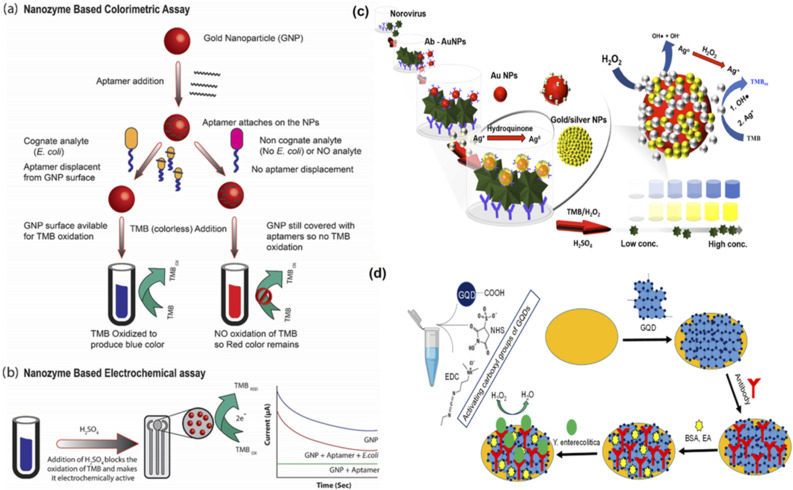
Nanozyme based sensors to detect pathogens in food. (a) NanoZyme colorimetric assay: In the presence of EC, the aptamer leaves the GNPs surface, enabling TMB oxidation and producing blue color. This is not the case in the absence of EC, the aptamers continue to cover the surface of GNPs, thus hindering the TMB oxidation, hence the color remains the same. (b) NanoZyme electrochemical assay: The TMB oxidation is blocked with H_2_SO_4_ which converts the oxidised TMB into electrochemically active entity. Thus, higher amount of oxidised TMB generates more current. Adapted with permission from ref. 85, copyright (2020) Elsevier. (c) Proposed silver-enhanced nanozyme-based immunoassay and silver-enhanced peroxidase-like activity of *in situ* Au/Ag NPs. Adapted with permission from ref. 105 copyright (2019) Elsevier. (d) Principle of the graphene quantum dots (GQDs)-based immunosensor for *Y. enterocolitica* detection. Adapted with permission from ref. 86, copyright (2019) Elsevier.

Several studies have been done recently that have used nanozymes to overcome any shortcomings based on enzyme-labelled antibodies during sensing of target analytes.^80,84,87,104^ For instance, Fu and colleagues developed a two-step cascade signal amplification of gold lateral flow assay using *in situ* gold growth and nanozyme catalysed deposition to detect *E. coli*.^81^ The researchers developed a unique, two-step cascade signal amplification strategy that combined both *in situ* growth of gold and nanozyme linked catalytic deposition to increase the detection sensitivity of the developed sensor. This nanozyme based sensing technique helped achieve a high LOD of 12.5 CFU mL^−1^, 400 times better results than the conventional methods. In another study by Das and colleagues, they developed an aptamer and nanozyme based electrochemical sensing strategy to detect *E. coli* within five minutes^85^ (Fig. 2). The group developed an aptamer-nanozyme-based assay based on the peroxidase-like activity of gold nanoparticles. These gold nanoparticles allowed the oxidation of 3,3′,5,5′-tetramethylbenzidine (TMB) to give a blue-coloured substrate. However, the aptamers also interact with the gold nanoparticles to reduce their peroxidase activity. Therefore, upon the presence of the target analyte, the aptamers interact with them instead of the gold nanoparticles. This way, the gold nanoparticles are turned on and are available for TMB oxidation. The sensor was noted to be rapid, highly sensitive, and inexpensive. Moreover, the results could be seen with naked eyes, and the LOD was recorded to be ∼10 CFU.

Another study by Savas and colleagues developed an immunosensor based on graphene quantum dots to detect *Yersinia enterecolitica* that causes yersiniosis in humans^86^ (Fig. 2). This bacterial infection results in the stomach and joint pain, diarrhoea, and fever in children and adults alike. This immunosensor could detect bacterial species in milk samples with high sensitivity without pretreatment and demonstrated a 5 CFU mL^−1^ LOD. Thus this nanozyme based immunosensor can also be used along with electrochemical biosensors for rapid and sensitive detection of different bacterial pathogens. Khoris and colleagues used the peroxidase activity of Au/AgNPs to detect norovirus^105^ The sensor functioned in two basic steps: interaction of gold probes with the target virus followed by increase in catalytic activity using silver ions (Fig. 2). The deposition of silver ions on the gold surface conferred enhanced affinity of the resultant nanozyme for peroxide molecules and resulted in an increased reaction rate with TMB owing to the more reactive species available. The sensor used anti-norovirus genogroup II antibodies and recorded a LOD of 13.2 copies per mL. In a closely related study by Weerathunge and colleagues, a nanozyme aptasensor was developed to perform the colourimetric detection of murine norovirus using gold nanoparticles.^106^ It could detect the target analyte with 30 viruses per mL LOD in a given sample in less than ten minutes and did not require expensive or sophisticated materials or equipment. Thus, such sensors have shown promising results in sensing pathogens in various food samples.

### 3.3. Antibiotics

Antibiotics are usually used either for treatment or as a preventive agent to keep animals and plants healthy; this is how they enter into the food chain. However, their presence in food is a matter of concern because they can cause serious health hazards upon accumulation. Therefore, their screening is necessary to ensure food safety. Though traditional methods like ELISA and HPLC are commonly used for this purpose, they are time-consuming and use expensive equipment. Therefore, alternate methods like nanozyme based biosensors are promising to carry out this process. For instance, a study by Zhang *et al.* used gold nanoclusters-based sensors that displayed peroxidase-like activity to detect tetracycline antibiotics using tetracycline-specific aptamers.^82^ The researchers exploited the inherent peroxidase-like activity of the gold nanoclusters and utilized the tetracycline aptamers to enhance the catalytic efficiency of the gold nanoclusters for TMB, the peroxidase substrates. The improved catalytic function in the presence of the aptamers allowed for sensitive and selective detection of the tetracycline molecules. The aptamers thus acted as the molecular recognition elements that allowed for specific attachment with the tetracycline, thus increasing the selectivity of the sensors. These sensors could detect the antibiotic with high specificity in the range of 1–16 μM and displayed a LOD of 46 nM. Similarly, Tian and colleagues used an electrochemical aptasensor to detect kanamycin using AuNO nanocomposites and CoFe_2_O_4_ nanozyme.^88^ This dual-mode sensor could detect between 1 pM to 1 μM and a LOD of 0.5 pM. This sensor used two nanozymes to carry out the assay: first colourimetric detection was done using the nanozyme to qualitatively and quantitatively the antibiotics followed by an electrochemical analysis using nanozymes that performed the catalysis of TMB to its oxidised form and therefore gave rapid and prominent results with high specificity and sensitivity towards the antibiotic. Thus, such sensors that permit double confirmation of the target analyte have a vast potential for food safety and analysis.

In another study by Zhang and colleagues, an aptamer labelled nanozyme based ELISA method was developed to detect ampicillin-linked BSA molecules in milk samples.^107^ This study made the use of AuNPs as nanozymes along with ampicillin/aptamer that acted as enzyme-labelled antibodies during the detection process. At the same time, the ampicillin conjugated BSA molecules functioned as coating antigens. The authors observed that as the number of ampicillin molecules increased, the conjugated Au-aptamer molecules levels also increased. Also, this method demonstrated high stability and high selectivity towards the target molecules and thus is a promising approach for food analysis. In another study by Youn *et al.*, a multiplexed sensor was developed to detect multiple antibiotics, including kanamycin, ampicillin, and sulfadimethoxine, using a fluorescence resonance energy transfer (FRET) method (FRET) with high specificity.^108^ The researchers used DNase I-assisted cyclic enzymatic signal amplification strategy and aptamer/graphene oxide molecules. The authors concluded that this method achieved a 2.1 times increase in signals during the detection process. The LOD was recorded as 1.997 ng mL^−1^ for sulfadimethoxine, 2.664 ng mL^−1^ for kanamycin, and 2.337 ng mL^−1^ for ampicillin molecules. Thus, this aptasensor allowed for simultaneous, rapid detection of multiple antibiotics with high specificity and massive potential for sensing different food biomarkers.

### 3.4. Hormones

Hormones like growth hormones are often used in animals like cows, fishes, and chickens to increase their growth in a short time. However, their overuse can cause adverse effects on our health, like cause metabolic disorders or abnormal body growth. For instance, 17β-estradiol is an estrogen steroid hormone that strongly disrupts the endocrine activity and is found in food samples. In a study by Yao and colleagues, a carboxyl modified Fe_3_O_4_ magnetic nanoparticles-based immunochromatography assay was developed to detect estradiol molecules.^83^ The team used carboxyl-modified Fe_3_O_4_ nanoparticles for labelling goat anti-mouse antibodies. Instead of the labelled monoclonal antibodies, freely available anti-E_2_ monoclonal antibodies were used to capture E_2_ molecules. The researchers confirmed that the excellent performance of the sensor was significant because of the lowered amount of free monoclonal antibodies and the strategy behind the signal amplification of the method. The sensor showed high sensitivity towards the bioanalyte and a 0.2 ng mL^−1^ LOD. Thus as the authors recorded, the LOD of the sensor was five times better than the conventional strips based on magnetic nanoparticles and twice better compared to the dual-probe strip. Also, this sensor successfully detected estradiol molecules from various food samples, including milk, fish, prawn, and chicken samples, and thus has promising applications towards sensing food biomarkers and food safety.

Likewise, Wang and coworkers developed a nanozyme with dual activity; that is, it functioned as a catalyst and luminescent sensor for the detection of 17β-estradiol and its derivatives.^109^ The nanozyme was developed from luminescent Tb^3+^ ions, hemin, and light harvesting ligand. The developed composite displayed highly stable, inexpensive, and displayed high catalytic activity. Furthermore, this synthesised nanozyme catalysed the degradation of 17β-estradiol and its derivatives during the detection process, and the LOD recorded was 50 pM. Thus, such nanozymes can be developed that display dual activity as a catalyst and a luminescent sensor, thus rapidly detecting the target analyte and promoting food safety.

### 3.5. Additives

Additives are added to many food items to improve their taste, texture, and smell; however, when used in large quantities, these result in severe health complications, including cancers. Detecting such additives is crucial to prevent long-term health complications and ensure food safety. Therefore, several studies have been done in the recent decade on developing nanozyme based biosensors for rapid detection of multiple additives like antioxidants, food colorants, sweeteners, preservatives from food samples.^89,90,110–117^

**Fig. 3:**
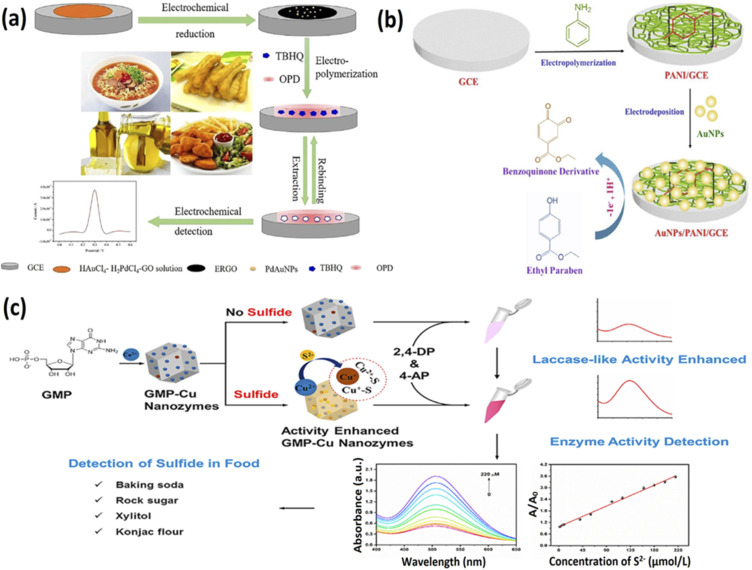
Nanozyme based sensors to detect additives in food. (a) The principle of constructing molecularly imprinted polymer-based sensor for tertiary butylhydroquinone detection. Adapted with permission from ref. 112 copyright (2019) Elsevier. (b) Construction of AuNPs/PANI/GCE interface to detect parabens in solubilised ionic liquid system. Adapted with permission from ref. 119, copyright (2020) Elsevier (c) Schematic representation of a novel selective detection method for sulfide in food systems based on the GMP-Cu nanozyme with laccase activity. Adapted with permission from ref. 91, copyright (2021) Elsevier.

Antioxidants are used in food to prevent them from spoiling because of rapid oxidation and therefore enhance food stability and shelf-life. However, the accumulation of antioxidants in the body may impair the immune system and malignancies, and hence their rapid detection is essential for food safety. Recently, several nanozyme based sensors have been developed for the rapid detection of antioxidants. For example, Ciu and colleagues a porphyrin-based porous organic polymer called the FePPOP-1 that demonstrated enhanced stability and peroxidase-like activity.^118^ They used this nanozyme in colourimetric detection of three different antioxidants, namely ascorbic acid, gallic acid, and tannic acid, and were successful at doing so. In another study by Yue *et al.*, an antioxidant, tertiary butylhydroquinone, was detected using an electrochemical sensor integrated with reduced graphene oxide, molecularly imprinted polymer, and Pd/AuNPs^112^ (Fig. 3). This sensor displayed high sensitivity towards the antioxidant and recorded a LOD of 0.046 μg mL^−1^ with a linear range of 0.5–60 μg mL^−1^.

Food colourants are synthetic colours added to food items to impart vibrant colours and are widely used in the food industry. Though the addition of colours to food and drinks gives them an aesthetically pleasing look, they are harmful when taken frequently and may result in toxicity in the human body. Sometimes, food colourants are used in certain ingredients to pass them as genuine products and increase the quantity of food. Thus, monitoring food colourants in different food items is necessary, and nanozyme based sensors offer great potential. In a study by Qin and colleagues, they developed a TiO_2_/electro-reduced graphene oxide nanocomposite-based electrochemical sensor to detect ponceau 4R and tartrazine.^89^ TiO_2_ NPs were attached to the electro-reduced graphene oxide molecules and provided the area to adsorb the target molecules and oxidise them. Upon their oxidation, peak currents were recorded by the sensor, and a linear increase in the current was observed upon increasing the concentration of the target analytes. The authors concluded that this inexpensive, simple sensor could rapidly detect the colourants within the range of 0.01–5 μM, and the LOD was noted to be 4 nM for ponceau 4R and 6 nM for tartrazine molecules. In addition, the authors noted the high selectivity, stability, and reproducibility of the sensor and could be used for on-site detection of various colourants. A closely similar study by Li and coworkers used the same nanozyme to develop a TiO_2_/ErGO/GCE-based sensor to carry out electrochemical detection of Allura Red in milk drinks and recorded a LOD of 0.05 μM.^90^ TiO_2_/ErGO was developed *via* titanium sulfate hydrolysis within graphene oxide suspension as well as *in situ* electrochemical reduction. It allowed for a facile and efficient pathway to develop nanohybrids with TiO_2_ nanoparticles coated with graphene nanoflakes. In another study by Li *et al.*, CuS nanoflowers were developed to perform the efficient electrochemical sensing of tartrazine and sunset yellow dyes.^114^ The researchers showed the morphology-controlled synthesis of CuS crystals *via* the facile hydrothermal/solvothermal method by altering the reaction solvents without using any surfactants, templates, or structure directing agents. The authors recorded a LOD of 0.012 μM for tartrazine and 0.006 μM for sunset yellow and thus demonstrated the high activity and potential of CuS nanoflowers towards electrochemical sensing of different colourants in food items and drinks.

Other food additives, like sweeteners and preservatives, enhance the texture, flavour, and durability of different food and drinks. Rather and colleagues developed an electrochemical sensor based on polyaniline (PANI) film, AuNPs, and glassy carbon electrodes to detect parabens^119^ (Fig. 3). This AuNPs/PANI/GCE interface showed an electrochemical response when endocrine disruptor ethylparaben molecules were oxidised in a solubilised ionic liquid cholinium chloride system. This sensor could detect the paraben molecules within the range of 0.1 nM to 5.10 nM and demonstrated a LOD of 0.1 nM towards the analyte. In another effort to analyse additives in food, Devi and coworkers developed an immunosensor based on AuNPs/molybdenum disulfide/chitosan nanocomposites to detect monosodium glutamate.^117^ The authors used a glassy carbon electrode modified with gold nanoparticles over a molybdenum disulfide/chitosan nanocomposite to develop the amperometric immunosensor. The developed nanocomposite functioned as a conductive matrix, and an anti-glutamate antibody was attached to its surface using the carbodiimide coupling method. The sensor displayed a detection range of 0.05–200 μM for the target molecule, and the LOD was noted to be 0.03 μM. Huang *et al.* used the laccase-like activity of GMP-Cu nanozymes to perform colourimetric detection of sulfide molecules^91^ (Fig. 3). The authors discovered that the presence of sulfide molecules increased the catalytic activity of the nanozymes by over 3.5 times and displayed a linear range of 0–220 μmol L^−1^ and a LOD of 0.67 μmol L^−1^ towards the detection of sulfide in food systems. The increased laccase activity was recorded for two reasons: (1) Reduction of Cu^2+^ present in the nanozyme to Cu^+^ and (2) the Cu–S bond formation that allowed the acceleration of electron transfer rate to enhance the catalytic efficiency. Hence, the protocol displayed an excellent selectivity for sulfide molecules. Thus these nanozyme based sensors can be successfully employed to regulate the various additives and ensure safety in the food industry. The Table 2 below provides an overview of the different nanozymes used towards the detection of different food biomarkers for food safety.

**Table 2:** Below sums up different nanozymes used to detect various food biomarkers

Nanozyme used	Enzyme like activity displayed	Food biomarker detected	Sample matrix	Detection range	Limit of detection	Ref.
SiO_2_/Au–Pt	Catalase	Aflatoxin B1	Blind peanut samples	0.01 ng mL^−1^ and 1000 ng mL^−1^	5 pg mL^−1^	120
AuNPs	Peroxidase	Aflatoxin B1	Feed samples	0.001–2 ng mL^−1^	0.453 pg mL^−1^	121
MIL-88	Peroxidase	Aflatoxin B1	Peanut milk and soy milk	0.01 to 20 ng mL^−1^	0.009 ng mL^−1^	122
Cu(OH)_2_ nanocages	Peroxidase	Microcystin-LR	Water samples	0.007 to 75 μg L^−1^	6 ng mL^−1^	123
AuNP-ICA	Peroxidase	*E. coli* O157:H7	Milk samples	1.25 × 10^1^ CFU mL^−1^ to 1.25 × 10^5^ CFU mL^−1^	1.25 × 10^1^ CFU mL^−1^	124
AuNPs	Peroxidase	*E. coli*	Fruit juice samples	10 to 10^9^ CFU mL^−1^	∼10 CFU	125
Graphene quantum dots	Peroxidase	*Yersinia enterocolitica*	Milk samples	1–6.23 × 10^8^ CFU mL^−1^	5 CFU mL^−1^	126
Au/Ag NPs	Peroxidase	Norovirus	—	1 pg mL^−1^–100 ng mL^−1^	10.8 pg mL^−1^	127
AuNPs	Peroxidase	Norovirus	—	1320–19 800 viruses per mL	50 viruses per mL	128
CoFe_2_O_4_	Peroxidase	Kanamycin	Milk samples	1 pM to 1 μM	0.5 pM	129
AuNCs	Peroxidase	Tetracycline	Milk samples	1–16 μM	46 nM	130
AuNPs	Peroxidase	Ampicillin	Milk samples	—	—	131
Tb-MOF	Peroxidase	17β-estradiol	—	0–100 nM	5 nM	132
Fe_3_O_4_ magnetic nanoparticles	Peroxidase	17β-estradiol	Milk, prawn, fish and chicken samples	0–0.5 ng mL^−1^	0.2 ng mL^−1^	133
GMP-Cu	Laccase	Sulfide	Baking soda, rock sugar, konjac flour and xylitol	0–220 μmol L^−1^	0.67 μmol L^−1^	134
MIP-PdAuNPs-ERGO/GCE	Peroxidase	Tertiary butylhydroquinone	Edible oils	0.5–60 μg mL^−1^	0.046 μg mL^−1^	135
CuS crystals	Peroxidase	Tartrazine and sunset yellow	Mirinda, jelly and candy	0.4–100 μM for tartrazine and 0.1–700 μM for sunset yellow	0.012 μM for tartrazine and 0.006 μM for sunset yellow	136

## 4. Challenges and possible solutions for the development of nanozyme based biosensors towards detection of various food biomarkers

As discussed in the previous sections, recently, nanozymes have shown some exciting potential in combating the persistent challenges in food safety. Nanozymes based sensors have paved a new pathway to develop novel, convenient, sensitive, efficient, and rapid analytical methods for detecting different food biomarkers to ensure food safety. However, a few challenges remain to be tackled for this emerging field to reach its full potential. For example, though nanozymes are great alternatives for natural enzymes, their catalytic activity is comparatively lower. Also, for colourimetric sensing approaches, the detection process can be affected by potential interference due to the background colour of the target molecules and give inaccurate results.^8^ Also, the specificity and the selectivity of nanozymes are not as strong as natural enzymes. Therefore, further works need to be done to improve the selectivity and sensitivity of nanozymes towards their target molecules. In addition, nanozyme based sensors like the surface-enhanced Raman spectroscopy sensors show lower selectivity in complex food samples because of the interference from several components like proteins. Such biomolecules may interfere during the biosensing process through adsorption to nanozymes, therefore changing their catalytic activity.

To avoid such interference and non-specific binding of target molecules during biosensing, nanozymes have been functionalised with bioreceptors including antibodies and aptamers that play a crucial role in enhancing the selectivity and sensitivity of the sensor.^32^ Furthermore, to enhance the overall activity of nanozyme based sensors, nanozymes can be functionalised with MOFs, silica, carbon, or hydrogels.^137–139^ With the modification of nanozymes with these groups, the surface area of the resultant complex increases, exposing more active sites and allowing for higher catalytic efficiency.^140^ Furthermore, organic ligands confer optical and electrical properties to nanozymes along with additional functional groups for chemical modifications. Single-atom nanozymes (SANS) are also a great option that allows utmost atomic utilisation. As a result, more active sites are available during the reaction, thus improving the overall activity during the sensing process.^141^ The activity of nanozyme based sensors can be enhanced such that they possess multiple modes of detection for different food biomarkers; for instance, electrochemical sensors, photoelectrochemical, piezoelectric sensors can be built on a common platform for accurate detection of food analytes. In addition, the selectivity is notably reduced when multiple food biomarkers are present in trace amounts in complex food items. Hence, more work needs to be done on nanozyme based sensors to increase stability, reproducibility, and sensitivity. Recent studies suggest that molecular imprinting technology offers a great solution to this issue.^142,143^ Linking nanozymes with molecularly imprinted polymers has improved the sensitivity and selectivity of the nanozymes and, thus, improved their overall activity during sensing.^144^ Furthermore, to enhance the efficiency of the sensing process and make the procedure more user-friendly, sensors can be integrated with smartphones to record, store, and share data related to the different food analytes detected. Also, as noted above, though a large number of studies have been conducted in recent years on nanozyme based sensors for food analysis, only a few have been further developed to be used as detection systems. Therefore, more work needs to be done to create rapid nanozyme based sensors integrated with digital colourimetric platforms that will help quick and efficient on-site screening of different food biomarkers to ensure food safety in the ever-evolving food industry.

## 5. Conclusions and future perspectives

Food safety is a rapidly emerging field that has gained widespread attention in the recent decade. Food safety and consumption is dependent on the amount of contaminants present in food. Not only do these contaminants pose a risk to human health, but they may also result in economic loss in food processing and availability.^145–147^ Therefore, developing effective and efficient strategies for contaminants or biomarkers associated with food quality/safety is imperative. So far, multiple conventional techniques have been developed to achieve this goal, for example, polymerase chain reaction,^148,149^ high-performance liquid chromatography,^150,151^ gas chromatography-mass spectrometry techniques,^152^*etc.* Though these methods allow for high sensitivity, precision, and reliability, they are cumbersome, complicated, require expensive instruments and trained professionals, and are also time-consuming. Therefore, these methods are not ideal for rapid and on-site detection of large amounts of samples. Moreover, these methods are difficult to be applied in underdeveloped countries and other poor localities.^8^ Thus, nanozyme based biosensors offer great solutions for detecting multiple food analytes.

In this review, we have summarised the recent studies done on different kinds of nanozyme based sensors. Though the conventional natural enzyme-based sensing processes display high sensitivity and sensitivity, they do not remain active for a long time and are expensive to develop. On the contrary, nanozymes, besides showing natural enzyme-like properties, are also more stable and cheaper. Therefore, using nanozymes in biosensors offer a great alternative in analysing food biomarkers and ensuring food safety. However, despite the promises this field holds, a few shortcomings still need to be addressed before their commercialisation and widespread availability in the food industry. As discussed in Section 5, the reproducibility of the results is still unclear. The nanozymes used during the sensing process are often developed in minute quantities, each in a separate laboratory using different techniques; thus, there is no uniformity in the nanozymes used, even if utilised for the detection process of the same analyte. This drawback can be solved by producing the required nanozyme in bulk on an industrial scale. Yet, another obstacle remains that needs to be addressed: recognition elements used during the experiments. Because different laboratories adopt unique protocols to carry out a similar investigation, the essential step of bioconjugation during biosensing differs, leading to a slightly different result.^153^ Therefore, standardised protocols must be developed to ensure the reproducibility of the results. In addition, manually analysing the visual signals as observed during colourimetric detection of food biomarkers sometimes results in recording incorrect data or may differ with individuals, resulting in uncertainty in the observed results. Therefore, combining digital technology with colourimetric detection techniques can give better observations. For instance, machine vision, similar to the human eye, can provide superior and easily quantifiable results and therefore help in the reduction of any potential errors during the manual study.^154^

Nanozymes are promising entities with considerable potential for detecting and monitoring different biomarkers in the food industry and thereby aid in the analysis of food contaminants. However, more studies need to be done to overcome the limitations mentioned above and further explore the application of nanozymes in the food industry.

## Author contributions

The manuscript was written through contributions of all authors. All authors have given approval to the final version of the manuscript.

## Conflicts of interest

There are no conflicts to declare.

## Supplementary Material
